# Comprehensive Cancer Control Partners’ Use of and Attitudes About Evidence-Based Practices

**DOI:** 10.5888/pcd12.150095

**Published:** 2015-07-16

**Authors:** C. Brooke Steele, John M. Rose, Julie S. Townsend, Jamila Fonseka, Lisa C. Richardson, Gary Chovnick

**Affiliations:** Author Affiliations: John M. Rose, Gary Chovnick, Health and Analytics, Battelle Memorial Institute, Arlington, Virginia, and Seattle, Washington; Julie S. Townsend, Jamila Fonseka, Lisa C. Richardson, Division of Cancer Prevention and Control, Centers for Disease Control and Prevention, Atlanta, Georgia.

## Abstract

**Introduction:**

National Comprehensive Cancer Control Program (NCCCP) awardees are encouraged to work with partners (eg, nonprofit organizations) to develop and implement plans to reduce the cancer burden in their jurisdictions using evidence-based practices (EBPs). However, the extent of EBP use among awardees and their partners is not well understood.

**Methods:**

From March through July 2012, we conducted a web-based survey of program partners referred by NCCCP program directors who were involved in implementation of cancer control plans.

**Results:**

Approximately 53% of referred partners (n = 83) completed surveys, 91.6% of whom represented organizations. Most partners reported involvement in helping to identify (80.5%), adapt (81.7%), implement (90.4%), and evaluate (81.9%) EBPs. The factors rated most frequently as very important when selecting EBPs were “consistent with our organization’s mission” (89.2%) and “cost-effective” (81.9%). Although most respondents said that their organizations understood the importance of using EBPs (84.3%) and had adequate access to cancer registry data (74.7%), few reported having sufficient financial resources to develop new EBPs (7.9%). The most frequently mentioned benefit of using EBPs was that they are proven to work. Resource limitations and difficulty adapting EBPs for specific populations and settings were challenges.

**Conclusions:**

Our findings help indicate how NCCCP partners are involved in using EBPs and can guide ongoing efforts to encourage the use of EBPs for cancer control. The challenges of using EBPs that partners identified highlight the need to improve strategies to translate cancer prevention and control research into practice in real-world settings and for diverse populations.

## Introduction

Comprehensive cancer control (CCC) is a collaborative process through which a community and its partners pool financial and nonfinancial resources to reduce the burden of cancer (1). The National Comprehensive Cancer Control Program (NCCCP), funded by the Centers for Disease Control and Prevention (CDC), provides technical support to CCC programs in 50 states, the District of Columbia, 7 US Associated Pacific Islands (USAPI)/territories, and 7 tribes or tribal organizations to develop, implement, and evaluate plans to prevent and control cancer in their jurisdictions. The NCCCP encourages CCC programs to work with coalitions, program stakeholders, or other partnerships to help conduct these activities (2). Partners may include health systems and nonprofit organizations and may be structured as part of health departments, 501(c)3 organizations, academic institutions, or cancer centers (3).

The NCCCP promotes the use of evidence-based practices (EBPs) for the development and implementation of cancer control plans and encourages grantees to use high-quality data and research to assess the burden of cancer in their jurisdictions, set priorities, and develop program goals (4–6). EBPs are defined as public health practices (interventions, programs, strategies, policies, procedures, processes, or activities) that have been tested or evaluated and shown to be effective; this definition is based on concepts of evidence-based public health (7–9). CCC partners have diverse skills and expertise that place them in a unique position to encourage the use of cancer-related EBPs (eg, cancer prevention and early detection interventions in the* Guide to Community Preventive Services*) by CCC programs (10,11). Many CCC partnerships include a core group of local organizations and individuals who provide coalition leadership and take responsibility for selected activities, a convening organization that coordinates activities and monitors the jurisdiction’s cancer burden, and a broad partnership that is dedicated to implementing the jurisdiction’s cancer control plan (11).

The extent of EBP use among CCC programs is not well understood, and little is known about the experiences of CCC programs and their partners in identifying and using EBPs (3,10,12,13). Potential barriers to the adoption of an EBP include characteristics of the practice as well as the organization that decides whether to adopt it (14,15). To address this gap, 2 surveys — one of CCC program directors and one of partners — were conducted to examine the use of scientific and practice-based information to inform development of cancer control plans and select evidence-based interventions. Findings from the survey of program directors have been published (16). The purposes of this study were to assess the partners’ perceptions of and roles in using EBPs for CCC with the aim of improving technical support to CCC programs related to identifying, adapting, implementing, and evaluating EBPs. This study was declared exempt from review by Battelle Memorial Institute’s institutional review board and was approved by the Office of Management and Budget (control no. 0920–0921).

## Methods

From March through July 2012, we conducted a cross-sectional survey targeting a convenience sample of up to 4 program partners referred by each of the CCC program directors who participated in the survey of directors. Program directors were asked to refer partners who were instrumental to the selection and implementation of cancer control EBPs in their jurisdictions. We included the 65 CCC programs that receive funding from CDC as well as CCC programs in 4 constituent states (Yap, Chuuk, Pohnpei, and Kosrae) supported by the national program in the Federated States of Micronesia. Therefore, partners of 69 programs were eligible to participate in this study. Recruitment for the partner survey began as soon as the program directors (or their designees) began providing their names and contact information. This information was entered into a survey tracking system, which was linked to the Web survey software and generated recruitment and reminder and follow-up email messages.

Survey measures were based on concepts from diffusion of innovations theory and dissemination science (14,17,18). Diffusion of innovations is defined as the process by which an innovation is communicated through certain channels over time among members of a social system (18). Characteristics of public health innovations that affect their rate of adoption include relative advantage, compatibility, complexity, and reinvention. We were also guided by a questionnaire developed by Hannon and colleagues to assess use of EBPs among members of the Cancer Prevention and Control Research Network (13). Our final variables and questionnaire items were designed to address use of EBPs and EBP resources (eg, the* Guide to Community Preventive Services*, Cancer Control P.L.A.N.E.T., Research-Tested Intervention Programs) (19–21), knowledge and attitudes about EBPs, characteristics of respondents and respondent organizations, and technical assistance needs of CCC program staff.

The Web-based, self-administered survey consisted of mostly close-ended, structured items. The questions were similar to those used in the questionnaire for program directors, but there were fewer total items. We used 2 open-ended questions to assess partners’ perceptions about benefits and challenges of using EBPs for cancer control. To understand how partners help CCC programs use EBPs, we asked about their involvement in 4 core activities related to using EBPs: identifying, adapting, implementing, and evaluating. To assess what characteristics or other factors are perceived as important when choosing EBPs in their jurisdictions and communities, we asked respondents to use a 4-point scale (very important, somewhat important, moderately important, not at all important) to rate the importance of a series of 14 items representing different aspects of EBPs. We also included questions intended to measure partners’ perceptions about their organizations’ capacities for using EBPs for cancer control. They were asked to indicate their level of agreement (strongly disagree, disagree, neither agree nor disagree, agree, strongly agree) with 11 statements representing organizational characteristics that could facilitate use of EBPs. To obtain feedback on the format and relevance of survey questions, as well as to estimate the time required to complete the survey, the questionnaire was pilot-tested with 6 partners referred by 3 program directors from a state, a tribe, and a territory. The questionnaire was modified based on findings from the pilot; therefore, the final analysis excludes pilot data. After excluding programs that piloted data (n = 3) and that opted not to participate in the study (n = 5) from the 69 programs eligible to participate, the total number of programs that referred partners was 61. The final instrument consisted of 40 questions. The partners received reminder emails and postcards if they did not complete their surveys.

Stata version 11 (StataCorp LP) was used for data analysis, including univariate analyses and exploratory bivariate analyses examining relationships between selected categorical variables via cross-tabulations. We used qualitative content analysis methods (22,23) using NVivo (version 9, QSR International) to examine key themes in the open-ended questions.

## Results

Of 158 partners who were referred by NCCCP program directors and sent invitations to participate in the survey, 83 completed surveys (response rate, 52.5%). The participating partners included 70 who were affiliated with state programs, 9 with USAPI/territories programs, and 4 with tribal or tribal organization programs. Most partners represented organizations, including voluntary health organizations, community-based or nonprofit organizations, health care systems, and local health departments. Nearly two-thirds of the respondents had graduate or professional degrees, and more than half had been working with the CCC programs in their jurisdictions for more than 5 years.

Most partners reported involvement in each of 4 core activities related to using EBPs (Table 1). The activity with the highest reported level of involvement was implementation of EBPs (90.4%); partners involved in identifying, adapting, and evaluating EBPs were nearly the same (80.5%–81.9%). Most partners were confident in their abilities to carry out these activities; approximately 90% were totally or somewhat confident that they could 1) assess whether certain EBPs are appropriate for use by the CCC programs in their jurisdictions, 2) find EBPs that could be used by the programs, and 3) adapt EBPs for specific populations or settings in their jurisdictions. Many were also confident that they could implement (83.1%) and evaluate (84.3%) EBPs.

**Table 1:** Respondent Characteristics, Survey of Uptake of Evidence-Based Practices Among National Comprehensive Cancer Control Program (NCCCP) Partners (n = 83), 2012^a^

Characteristic	n (%)
**Involved with CCC grantee as**	
Representative of an organization	76 (91.6)
Individual	7 (8.4)
**Highest level of education completed**	
High school graduate/GED	1 (1.2)
Technical or vocational school	1 (1.2)
Some college	3 (3.6)
College graduate	24 (28.9)
Graduate or professional degree	54 (65.1)
**Graduate/professional degree in public health/medical field**	
Yes	44 (81.5)
**Length of time working with CCC program, y**	
<1	1 (1.2)
1–3	17 (20.5)
4 or 5	18 (21.7)
>5	47 (56.6)
**Core activity for using EBPs with CCC program**	
Identifying interventions	66 (80.5)
Adapting interventions	67 (81.7)
Implementing interventions	75 (90.4)
Evaluating interventions	68 (81.9)
**Totally or somewhat confident in personal ability to . . .**	
Assess appropriateness of EBPs for your jurisdiction	76 (91.6)
Find potential EBPs for CCC program in your jurisdiction	75 (90.4)
Adapt EBPs for specific populations or settings	74 (89.2)
Evaluate implementation and effectiveness of EBPs	70 (84.3)
Implement EBPs with fidelity	69 (83.1)

Abbreviations: CCC, comprehensive cancer control; EBPs, evidence-based practices; GED, general equivalency development.

a Because of missing data or nonresponse, denominators for some categories did not total 83.

The characteristics rated most frequently as very important when choosing EBPs were “consistent with our organization’s mission” (89.2%), “cost-effective” (81.9%), “scientific evidence indicates EBPs work” (78.3%), and “easy to implement” (72.3%) (Table 2). Overall, most respondents strongly agreed or agreed with most statements about organizational characteristics that could encourage EBP use, including “my CCC organization understands the importance of using EBPs” (84.3%), “my CCC organization has adequate access to cancer registry data” (74.7%), and “using EBPs is part of the norm in my CCC organization” (74.7%) (Table 3). A much smaller percentage of partners strongly agreed or agreed that their organizations had sufficient staffing to develop new EBPs (27.7%) or financial resources for both the development of new EBPs (7.9%) and implementation of existing EBPs (23.7%).

**Table 2:** Characteristics Rated as Very Important When Selecting Evidence-Based Practices (EBPs) **a** Among National Comprehensive Cancer Control Program (NCCCP) Partners (n = 83), 2012^b^

Characteristic	n (%)
Consistent with our organization’s mission	74 (89.2)
Cost-effective	68 (81.9)
Scientific evidence saying it works	65 (78.3)
Easy to implement	60 (72.3)
Available for free or low cost	57 (68.7)
Easy to evaluate	52 (62.7)
Easily adaptable	51 (61.4)
Encouraged by funders	36 (43.4)
Other organizations like ours are using it	33 (40.2)
People in our community requested it	32 (38.6)
Technical assistance is available	26 (31.3)
Innovative	22 (26.8)
We had used it before	11 (13.3)
Lack of alternatives	9 (10.8)

a The question was, “In general, how important are each of the following factors to your CCC program when choosing an EBP?”

b Because of missing data or nonresponse, denominators for some categories did not total 83.

**Table 3:** Perceived Organizational Capacity for Using Evidence-based Practices^a^ Among National Comprehensive Cancer Control Program Partners (n = 83), 2012^b^

Strongly agreed or agreed with statement	n (%)
My CCC organization understands the importance of using EBPs	64 (84.3)
My CCC organization has adequate access to cancer registry data	56 (74.7)
Using EBPs is part of the norm in my CCC organization	56 (74.7)
My CCC organization has community members who can advise us on EBPs	54 (71.0)
My CCC organization has sufficient access to epidemiological expertise	52 (68.4)
My CCC organization has sufficient data support to inform our cancer control planning	51 (67.1)
My CCC organization has a champion who supports the use of EBPs	51 (67.1)
My CCC organization has sufficient staff in place to implement EBPs	37 (48.7)
My CCC organization has sufficient staff in place to develop new EBPs	21 (27.7)
My CCC organization has sufficient financial resources to implement existing EBPs	18 (23.7)
My CCC organization has sufficient financial resources to develop new EBPs	6 (7.9)

Abbreviations: CCC, comprehensive cancer control; EBP, evidence-based practice.

a The question was, “Please indicate your level of agreement with the following statements.”

b Because of missing data or nonresponse, denominators for some categories did not total 83.

We grouped the most frequently reported benefits of using EBPs into the following categories: proven to work, measurability, cost-effectiveness, credibility with funders and coalitions, recommended by funders, provide community benefits, and availability of technical assistance. The most frequently mentioned benefit of using EBPs was that they are proven to work, which some respondents associated with increasing the likelihood of successful outcomes. Many respondents said that EBPs provide a scientific basis for measuring and evaluating program impact, are replicable, and can be adapted. Some respondents noted that using EBPs is an efficient use of resources because programs do not have to spend funds developing and testing interventions. Other perceived benefits were that EBPs come with technical assistance, and their use increases the credibility of CCC programs among community partners and sponsoring agencies.

We also grouped perceived challenges of using EBPs into several categories, including resource limitations; lack of appropriate EBPs for specific populations and communities; adapting, implementing, and evaluating EBPs; and obtaining community buy-in. Most respondents identified resource limitations as a barrier, including lack of funds to develop and test interventions and adapt materials. Some respondents who said that EBPs may not be a good fit for specific populations noted that sufficient evidence was lacking for these groups because the EBPs had not been field-tested in their communities. Barriers related to adapting EBPs included uncertainty about maintaining fidelity when evidence-based interventions are implemented, lack of technical support, and difficulties tailoring interventions for populations that may not “trust government or science.” A few respondents said that implementing and evaluating EBPs were challenging and that multicomponent interventions were costly. Others commented that evaluating and measuring outcomes for EBPs that had been tested in research rather than real-world settings was difficult. One respondent noted that it would be useful to have logic models for EBPs that list resources needed, key activities, and evaluation steps.

## Discussion

This is the first study to comprehensively and specifically assess perceptions about EBPs among selected partners of NCCCP-funded programs, as well as their roles and responsibilities in using EBPs for developing and implementing cancer control plans. In 2009, NCCCP programs reported that they were working in partnership with more than 6,000 organizations and individuals, including government public health programs (94%), academic (94%) and professional (84%) organizations, cancer survivors (63%), and political leaders (51%) (11). The programs often leverage the resources of these partners (eg, donated time, financial support, subject matter expertise) to develop and implement interventions in cancer control plans (24); therefore, CCC partners are crucial to accelerating the adoption of EBPs.

More than 90% of partners reported involvement in implementing EBPs. This percentage is higher than the level of partner involvement in implementing interventions reported by NCCCP program directors in previous studies. Townsend and colleagues evaluated performance measures data reported by NCCCP awardees to CDC as a condition of their funding (24). In their analysis, 60% of programs reported that their partners had implemented at least one priority strategy from their jurisdiction’s cancer control plan from June 2008 through June 2009. Rochester and colleagues, in their pilot test of a 2007 NCCCP grantee performance measurement worksheet, also reported that 60% of programs indicated that their partners had implemented interventions (25). These discrepancies may be related to the fact that we asked CCC program directors to refer partners who collaborate with them in the selection and use of EBPs, thus providing us with a sampling pool more likely to include partners involved with implementation. Additionally, the Townsend and Rochester studies assessed all interventions reported by programs, not just those that were evidence-based. The differences in findings could also reflect an increase in partner involvement with EBP implementation over time — our survey was conducted more recently, and the performance measures for the current NCCCP project period (2012–2017) place a greater emphasis on collaborating with partners by forming coalition and partner workgroups to implement interventions and coordinate with other chronic disease prevention programs (24).

The percentages of partners who rated various characteristics as very important in the selection of EBPs are similar to the percentages of NCCCP program directors who selected this rating for the same characteristics (16). These rankings are also similar to those reported by cancer control planners who participated in a study by Hannon and colleagues (13). In that study, participants said consistency with their organization’s mission, cost-effectiveness, and evidence for the overall effectiveness of a program were important in selecting evidence-based programs. Many of these factors (eg, cost-effectiveness, ease of implementation, compatibility with an organization’s mission) have been reported in some translation and dissemination studies to predict successful adoption of EBPs (14,26). However, the importance of these attributes in the use of EBPs may vary by setting (14). Viewed through the lenses of diffusion of innovations theory and dissemination science, the interaction among intervention characteristics, the intended adopters, and organizational context or features determine adoption and implementation of EBPs (14,15).

We found substantial differences in perceptions about organizational capacity for using EBPs. Although most respondents indicated that their organizations had sufficient access to cancer surveillance data, community advisors, and epidemiologic support, many reported limited financial and staffing resources. Some CCC partner organizations receive financial assistance from CCC programs to help implement cancer plans; nearly one-third of NCCCP program directors reported that they provide funding and other support to their partners for CCC activities (16). The CCC National Partnership, a collaborative group of diverse national organizations, also provides financial resources to CCC coalitions and partnerships (27). Collaboration and leveraging shared resources are embodied in the definition of CCC, and both are critical to sustaining efforts by communities and their partners to reduce the burden of cancer.

The benefits of using EBPs that the partners identified were similar to those reported by CCC program directors (16). The most frequently reported benefit for both groups was that EBPs have been proven to work. Many also indicated that EBPs are measurable. Systematic reviews of evidence-based interventions to prevent and control chronic disease are available at no cost to cancer control planners on Web portals such as Cancer Control P.L.A.N.E.T (20). Because evidence gaps remain for some strategies, the reviews are updated periodically to identify new studies and determine whether additional evidence would change existing recommendations (28). Partners and program directors identified similar challenges to using EBPs, including limited interventions for specific populations and difficulty adapting EBPs (16). The latter finding was unexpected, because 89% of partners said they were totally or somewhat confident in their personal abilities to adapt EBPs. Fewer were confident in their abilities to implement EBPs with fidelity, which may be an aspect of adapting EBPs that some find challenging. Partners also were less confident in evaluating the implementation and effectiveness of EBPs. To help address these knowledge gaps, CDC, the National Cancer Institute, the CCC National Partnership, and other cancer control entities have developed webinars, trainings, and tools to help cancer control planners move research into practice (10,27). Additional training may be needed on selecting an EBP on the basis of its fit for a community and strength of evidence; on adhering to set protocols, core elements, and process steps for an EBP to maintain fidelity; and on evaluating interventions and campaigns that have been adapted.

In addition to the findings presented here, the contributions of state, tribal, and territorial CCC coalitions and partnerships in creating and implementing cancer control plans have been reported (3,10,12,13,28,29). Fewer studies have been published about partner involvement in EBP evaluation; thus, additional research is needed in this area (10,30). Evaluation studies are crucial to building the evidence base for EBPs.

This study has at least 3 limitations. First, only 53% of referred CCC partners participated. The partners were affiliated with various entities (eg, national health organizations, community-based organizations, health care providers, health departments, academic medical centers), but some groups may not have been represented. Second, pilot testing a survey may not adequately verify its validity and reproducibility. Third, limiting our sample to partners involved in implementing cancer control plans may have introduced a selection bias that affected the rating of the survey questions. We were interested in this subset of coalition members because they could respond to questions about using cancer-related interventions on the basis of their experience. Social desirability bias also may have been introduced if respondents did not answer questions according to their true beliefs. However, our results help expand our understanding of CCC partners’ roles in using EBPs and can guide ongoing efforts to promote their use and dissemination for cancer control.

CCC encompasses the cancer continuum (eg, prevention, early detection and diagnosis, treatment, survivorship, end of life); therefore, the context of cancer control (public health, primary care, specialty care) influences the mechanisms by which EBPs are disseminated and implemented (15). Public health and clinical communities were represented in our study. Their perspectives on the benefits of using EBPs support the continued use of evidence-based approaches to guide efforts to reduce the burden of cancer. The challenges of using EBPs that they identified highlight the need to improve strategies to translate cancer prevention and control research into practice in real-world settings and for diverse populations.

## References

[1] Centers for Disease Control and Prevention. National Comprehensive Cancer Control Program; 2014. http://www.cdc.gov/cancer/ncccp/. Accessed February 3, 2014.

[2] Major A , Stewart SL . Celebrating 10 years of the National Comprehensive Cancer Control Program, 1998 to 2008. Prev Chronic Dis 2009;6(4):A133. 19755009 PMC2774647

[3] Behringer B , Lofton S , Knight ML . Models for local implementation of comprehensive cancer control: meeting local cancer control needs through community collaboration. Cancer Causes Control 2010;21(12):1995–2004. 10.1007/s10552-010-9655-x 20938731

[4] Abed J , Reilley B , Butler MO , Kean T , Wong F , Hohman K . Developing a framework for comprehensive cancer prevention and control in the United States: an initiative of the Centers for Disease Control and Prevention. J Public Health Manag Pract 2000;6(2):67–78. 10.1097/00124784-200006020-00011 10787781

[5] Abed J , Reilley B , Butler MO , Kean T , Wong F , Hohman K . Comprehensive cancer control initiative of the Centers for Disease Control and Prevention: an example of participatory innovation diffusion. J Public Health Manag Pract 2000;6(2):79–92. 10.1097/00124784-200006020-00012 10787782

[6] Centers for Disease Control and Prevention. Guidance for comprehensive cancer control planning. Atlanta (GA): Department of Health and Human Services; 2002.

[7] Kerner JF , Hall KL . Research dissemination and diffusion: translation within science and society. Res Soc Work Pract 2009;19(5):519–30. 10.1177/1049731509335585

[8] Brownson RC , Gurney JG , Land GH . Evidence-based decision making in public health. J Public Health Manag Pract 1999;5(5):86–97. 10.1097/00124784-199909000-00012 10558389

[9] Kohatsu ND , Robinson JG , Torner JC . Evidence-based public health: an evolving concept. Am J Prev Med 2004;27(5):417–21. 15556743 10.1016/j.amepre.2004.07.019

[10] Vinson C , La Porta M , Todd W , Palafox NA , Wilson KM , Fairley T . Research and comprehensive cancer control coalitions. Cancer Causes Control 2010;21(12):2033–40. 10.1007/s10552-010-9667-6 21046447

[11] Rochester PW , Townsend JS , Given L , Krebill H , Balderrama S , Vinson C . Comprehensive cancer control: progress and accomplishments. Cancer Causes Control 2010;21(12):1967–77. 10.1007/s10552-010-9657-8 21069448

[12] Seeff LC , Major A , Townsend JS , Provost E , Redwood D , Espey D , Comprehensive cancer control programs and coalitions: partnering to launch successful colorectal cancer screening initiatives. Cancer Causes Control 2010;21(12):2023–31. 10.1007/s10552-010-9664-9 21086035

[13] Hannon PA , Fernandez ME , Williams RS , Mullen PD , Escoffery C , Kreuter MW , Cancer control planners’ perceptions and use of evidence-based programs. J Public Health Manag Pract 2010;16(3):E1–8. 10.1097/PHH.0b013e3181b3a3b1 20357600 PMC2920604

[14] Greenhalgh T , Robert G , Macfarlane F , Bate P , Kyriakidou O . Diffusion of innovations in service organizations: systematic review and recommendations. Milbank Q 2004;82(4):581–629. 10.1111/j.0887-378X.2004.00325.x 15595944 PMC2690184

[15] Kerner JF , Guirguis-Blake J , Hennessy KD , Brounstein PJ , Vinson C , Schwartz RH , Translating research into improved outcomes in comprehensive cancer control. Cancer Causes Control 2005;16(S1, Suppl 1):27–40. 10.1007/s10552-005-0488-y 16208572

[16] Steele CB , Rose JM , Chovnick G , Townsend JS , Stockmyer CK , Use of evidence-based practices and resources among comprehensive cancer control programs. J Public Health Manag Pract 2014 Jan 7 [Epub ahead of print]. 10.1007/s10552-005-0488-y 24402431 PMC4620697

[17] Rabin BA , Glasgow RE , Kerner JF , Klump MP , Brownson RC . Dissemination and implementation research on community-based cancer prevention: a systematic review. Am J Prev Med 2010;38(4):443–56. 20307814 10.1016/j.amepre.2009.12.035

[18] Rogers EM . Diffusion of innovations. 5th edition. New York (NY): Free Press; 2003.

[19] Guide to Commmunity Preventive Services. http//www.thecommunityguide.org/index.html. Accessed August 13, 2010.

[20] Cancer Control P.L.A.N.E.T . http//cancercontrolplanet.cancer.gov/index/html. Accessed August 13, 2010.

[21] Research-Tested Intervention Programs (RTIPs). http://rtips.cancer.gov/rtips. Accessed August 3, 2010.

[22] Morgan DL . Qualitative content analysis: a guide to paths not taken. Qual Health Res 1993;3(1):112–21. 10.1177/104973239300300107 8457790

[23] Patton MQ . Qualitative Evaluation and Research Methods. 2nd edition. Newbury Park (CA): Sage Publications; 1990.

[24] Townsend JS , Moore AR , Mulder TN , Boyd M . What does a performance measurement system tell us about the National Comprehensive Cancer Control Program? J Public Health Manag Pract 2014;00(00):1–8 [Published online 2014 Aug 18]. 10.1097/PHH.0000000000000124 25136936 PMC4374017

[25] Rochester P , Porterfield DS , Richardson LC , McAleer K , Adams E , Holden D . Piloting performance measurement for Comprehensive Cancer Control programs. J Public Health Manag Pract 2011;17(3):275–82. 10.1097/PHH.0b013e3181fd4d19 21464689

[26] Pollack LA , Hawkins NA , Peaker BL , Buchanan N , Risendal BC . Dissemination and translation: a frontier for cancer survivorship research. Cancer Epidemiol Biomarkers Prev 2011;20(10):2093–8. 10.1158/1055-9965.EPI-11-0652 21980017 PMC4890627

[27] Hohman K , Rochester P , Kean T , Belle-Isle L . The CCC National Partnership: an example of organizations collaborating on comprehensive cancer control. Cancer Causes Control 2010;21(12):1979–85. 10.1007/s10552-010-9644-0 20872239

[28] Sabatino SA , Lawrence B , Elder R , Mercer SL , Wilson KM , DeVinney B , Effectiveness of interventions to increase screening for breast, cervical, and colorectal cancers: nine updated systematic reviews for the guide to community preventive services. Am J Prev Med 2012;43(1):97–118. 10.1016/j.amepre.2012.04.009 22704754

[29] Weinberg AD , Jackson PM , DeCourtney CA , Cravatt K , Ogo J , Sanchez MM , Progress in addressing disparities through comprehensive cancer control. Cancer Causes Control 2010;21(12):2015–21. 10.1007/s10552-010-9649-8 21057869

[30] Alberg AJ , Cartmell KB , Sterba KR , Bolick S , Daguise VG , Hébert JR . Outcome evaluation of a state comprehensive cancer control plan: laying the foundation. J Public Health Manag Pract 2013;19(4):300–7. 10.1097/PHH.0b013e31825d208c 23381113 PMC3777627

